# β,β-Dimethylacrylalkannin, a Natural Naphthoquinone, Inhibits the Growth of Hepatocellular Carcinoma Cells by Modulating Tumor-Associated Macrophages

**DOI:** 10.3390/molecules29163919

**Published:** 2024-08-20

**Authors:** Li-Sha Shen, Zesi Lin, Rui-Hong Gong, Yu-Shan Lin, Xing-Fang Qiao, Qian-Mei Hu, Wei-Han Qin, Sibao Chen, Yong Yang, Guo-Qing Chen

**Affiliations:** 1 Chongqing Academy of Chinese Materia Medica, Chongqing 400065, Chinayangychem@126.com (Y.Y.); 2 Sichuan–Chongqing Joint Key Laboratory of Innovation of New Drugs of Traditional Chinese Medicine, Chongqing 400065, China; 3 Southern Medical University of Hospital of Integrated Traditional Chinese Medicine and Western Medicine, Southern Medical University, Guangzhou 510515, China; 4 Department of Food Science and Nutrition, The Hong Kong Polytechnic University, Hung Hom, Hong Kong 999077, Chinasibao.chen@polyu.edu.hk (S.C.); 5 State Key Laboratory of Chinese Medicine and Molecular Pharmacology (Incubation), The Hong Kong Polytechnic University Shenzhen Research Institute, Shenzhen 518057, China; 6 Research Centre for Chinese Medicine Innovation, The Hong Kong Polytechnic University, Hung Hom, Hong Kong 999077, China

**Keywords:** β,β-dimethylacrylalkannin, hepatocellular carcinoma, macrophage polarization, THP-1, co-culture

## Abstract

Tumor-associated macrophages (TAMs) are pivotal in the tumor microenvironment (TME) of hepatocellular carcinoma (HCC), influencing various stages from initiation to metastasis. Understanding the role of TAMs in HCC is crucial for developing novel therapeutic strategies. Macrophages exhibit plasticity, resulting in M1 and M2 phenotypes, with M1 macrophages displaying antitumor properties and M2 macrophages promoting tumor progression. Targeting TAMs to alter their polarization could offer new avenues for HCC treatment. β,β-dimethylacrylalkannin (DMAKN), a natural naphthoquinone, has gained attention for its antitumor properties. However, its impact on TAMs modulation remains unclear. This study investigates DMAKN’s modulation of TAMs and its anti-HCC activity. Using an in vitro model with THP-1 cells, we induced M1 macrophages with LPS/IFN-γ and M2 macrophages with IL-4/IL-13, confirming polarization with specific markers. Co-culturing these macrophages with HCC cells showed that M1 cells inhibited HCC growth, while M2 cells promoted it. Screening for non-toxic DMAKN concentrations revealed its ability to induce M1 polarization and enhance LPS/IFN-γ-induced M1 macrophages, both showing anti-HCC effects. Conversely, DMAKN suppressed IL-4/IL-13-induced M2 polarization, inhibiting M2 macrophages’ promotion of HCC cell viability. In summary, DMAKN induces and enhances M1 polarization while inhibiting M2 polarization of macrophages, thereby inhibiting HCC cell growth. These findings suggest that DMAKN has the potential to regulate TAMs in HCC, offering promise for future therapeutic development.

## 1. Introduction

Hepatocellular carcinoma (HCC), the most common form of liver cancer, is a leading cause of cancer-related mortality worldwide [1]. Risk factors for HCC development include viral infections, excessive alcohol consumption, drug induction, obesity, diabetes, cholestasis, and genetic factors [2]. Despite advances in diagnosis and treatment, the incidence and mortality rates of HCC continue to rise. This increase is due to the complexity of its molecular mechanisms, high intratumor heterogeneity, and multiple treatment resistances, resulting in persistently low survival rates [3].

Recent studies have highlighted the critical role of the tumor microenvironment (TME) in HCC occurrence and progression [4]. The interaction between HCC cells and the TME is essential for tumor growth and development [5]. The TME causes HCC cells to acquire abnormal phenotypes and recruit immune cells, with tumor-associated macrophages (TAMs) being key players in establishing and maintaining the TME [6].

TAMs develop a unique phenotype within the TME, promoting tumorigenesis, development, invasion, and metastasis, particularly in tumor blood vessels and lymphatics. Macrophages are categorized as classically activated macrophages (M1) and alternatively activated macrophages (M2). TAMs play a dual role in tumor development, where M1 macrophages kill tumor cells, while M2 macrophages promote tumor growth [7]. In the TME, TAMs predominantly exhibit an M2 phenotype [8]. In HCC, significant macrophage infiltration in the TME is often associated with poor prognosis. The polarization and infiltration of TAMs are crucial mechanisms in HCC development. TAMs contribute to tumor progression by modulating the immune response and secreting various cytokines [9,10]. Consequently, targeting TAMs may offer new therapeutic strategies for HCC patients and has become a focus of emerging research [11].

Natural products are a valuable source of antitumor agents, offering structural diversity, abundant resources, and low side effects [12]. Recent research has focused on the role of natural products in modulating the TME. Numerous reports have suggested that natural compounds have a strong potential to affect immunity by targeting TAMs polarization and infiltration, thereby improving the TME and exerting antitumor effects [13].

**Figure 1:**
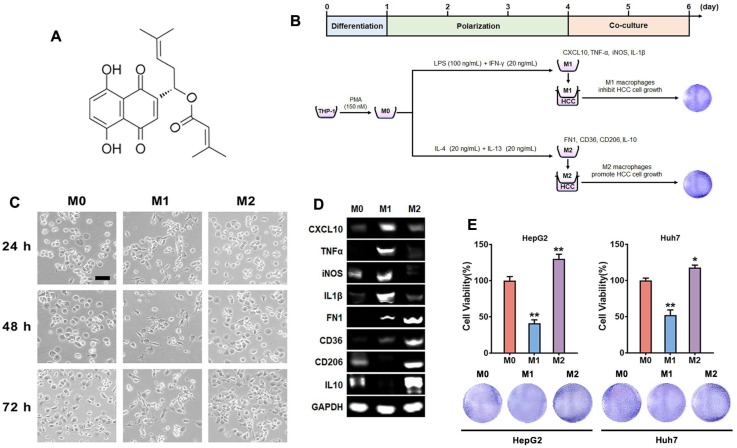
Polarization of THP-1 macrophages and their effects on HCC cells. (**A**) Chemical structure of DMAKN. (**B**) Differentiation of THP-1 cells into macrophages, subsequent polarization into M1 or M2, and co-culture with HCC cells. (**C**) Morphological characteristics of M0, M1, and M2 macrophages. Scale bar = 50 μm. (**D**) mRNA expression of M1 and M2 markers. (**E**) Impact of macrophages co-cultured with HCC cells. upper panel: cell viability of HCC cells co-cultured with macrophages. lower panel: visualization of formed formazan crystals in co-cultured plates. * *p* < 0.05, ** *p* < 0.01, compared with M0 cells.

β,β-dimethylacrylalkannin (DMAKN), a natural naphthoquinone depicted in Figure 1A, has shown significant antitumor activity [14]. Importantly, it demonstrated potential safety in oral acute and chronic toxicity studies, supporting further development as a therapeutic agent [15]. However, it remains unclear whether this compound can regulate macrophage polarization and exert antitumor activity through TAMs modulation. Therefore, our study aims to investigate DMAKN’s ability to modulate macrophage polarization and its anti-HCC activity through TAMs modulation.

## 2. Results

### 2.1. Polarizing THP-1 Macrophages and Their Effects on HCC Cells

In this study, THP-1 cells, a human monocytic cell line, were used to investigate macrophage polarization and its impact on HCC cell viability [16]. The differentiation of THP-1 monocytes into macrophages (M0) and their polarization into TAMs is shown in Figure 1B. Initially, THP-1 monocytes were cultured with PMA for 1 d to induce differentiation into M0 macrophages. To polarize the M0 macrophages into the M1 phenotype, LPS and IFN-γ were added for 3 d. Simultaneously, IL-4 and IL-13 were used to polarize M0 macrophages into the M2 phenotype. After 3 d, morphological differences among M0, M1, and M2 macrophages were evident. Figure 1C shows M0 cells as predominantly round or oval, M1 cells as short, spreading, vacuolated, and with a flat round shape, and M2 cells as elongated.

We also screened specific markers for the M1 and M2 phenotypes in our models using THP1 monocytes. Figure 1D confirms the increased mRNA expression of M1 markers (CXCL10, TNF-α, iNOS, IL-1β) following differentiation into the M1 phenotype. Similarly, M2 markers (FN1, CD36, CD206, IL-10) showed increased mRNA expression upon differentiation into the M2 phenotype. These results validate our protocol for differentiating THP-1 monocytes into macrophages and polarizing them into M1 and M2 types.

Next, we assessed the impact of M1 and M2 macrophages on HepG2 and Huh7, two HCC cell lines. Following polarization, the macrophages were co-cultured with HCC cells at a 5:1 ratio for 2 d. Cell viability was then recorded. As expected, the results in Figure 1E indicate that the co-culture with M1 macrophages significantly decreased the viability of HCC cells. In contrast, the co-culture with M2 macrophages increased cell viability compared to the co-culture with M0 macrophages. These findings highlight that M1 polarized macrophages exhibit an anti-HCC effect, whereas M2 polarized macrophages support HCC cell viability.

### 2.2. Screening DMAKN Concentrations Used in Macrophages

**Figure 2:**
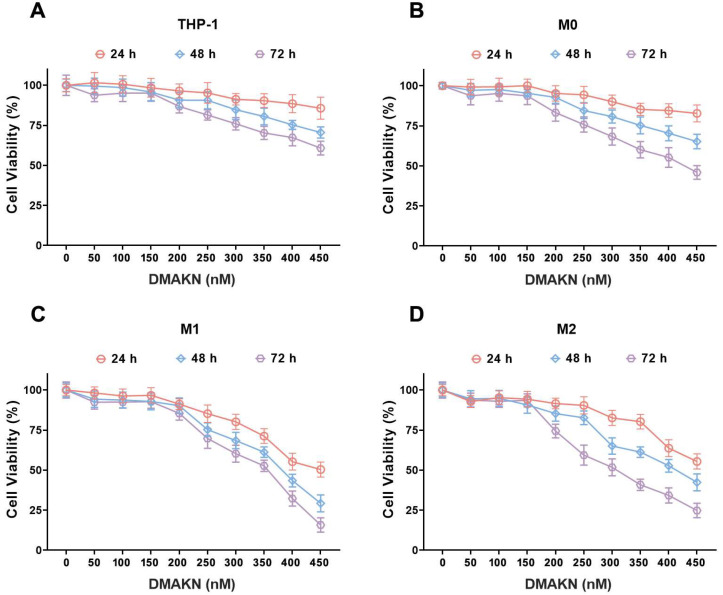
Screening the concentrations of DMKAN used in macrophages. (**A**)THP-1 cells, (**B**) M0 macrophages, (**C**) M1 macrophages, and (**D**) M2 macrophages were treated with the indicated concentration of DMAKN, and their cell viability was assessed using MTT separately at 24 h, 48 h, and 72 h.

Before assessing DMAKN’s impact on macrophage polarization, its effect on the viability of THP-1 monocytic cells and differentiated macrophages was screened. Various DMAKN concentrations (50, 100, 150, 200, 250, 300, 350, 400, and 450 nM) were tested. As shown in Figure 2A, DMAKN concentrations above 150 nM significantly reduced THP-1 cell viability, with greater effects observed at higher concentrations and longer durations. Similarly, Figure 2B–D illustrate that DMAKN concentrations at or below 150 nM did not affect cell viability in differentiated M0, M1, and M2 macrophages after 72 h of treatment. Therefore, DMAKN concentrations up to 150 nM were deemed non-toxic to macrophages and were used in the subsequent experiments (50, 100, 150 nM).

### 2.3. DMAKN-Induced M1 Macrophage Polarization Exhibits Anti-HCC Effects

**Figure 3:**
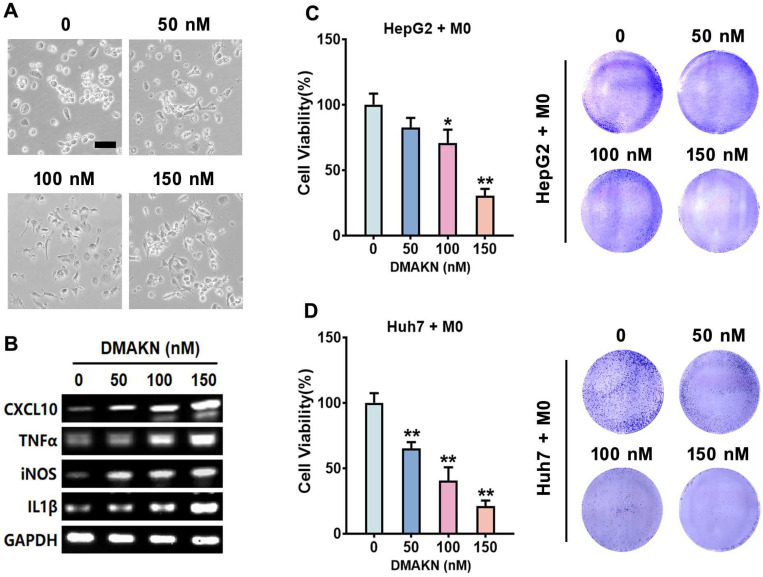
DMAKN-induced M1 macrophage polarization. (**A**) Morphological characteristics of DMAKN-induced M1 macrophage polarization. Scale bar = 50 μm. (**B**) mRNA expression of M1 markers in DMAKN-induced M1 macrophages. (**C**) Co-culture of DMAKN-induced M1 macrophages with HepG2 and (**D**) Huh7 cells. left panel: measurement of HCC cell viability. right panel: visualization of formed formazan crystals in co-cultured plates. * *p* < 0.05, ** *p* < 0.01, compared with M0 cells without DMAKN.

We investigated DMAKN’s impact on macrophage polarization using non-toxic concentrations (50, 100, and 150 nM). After THP-1 monocytes differentiated into M0 macrophages, DMAKN was added to the culture medium for 3 d. Figure 3A illustrates morphological changes resembling M1 macrophages, suggesting DMAKN induces M1 polarization. To confirm the DMAKN-induced M1 polarization, we analyzed M1 markers expression. Figure 3B shows the upregulation of these M1 markers, validating M1 polarization.

Next, we co-cultured DMAKN-induced M1 macrophages with HCC cells for 48 h. Figure 3C,D indicate reduced HCC cell viability, with higher DMAKN concentrations leading to greater reductions. These results demonstrate DMAKN’s ability to induce M1 macrophage polarization, which exerts anti-HCC effects.

### 2.4. DMAKN Enhances Anti-HCC Activity by Sensitizing M1 Macrophage Polarization

**Figure 4:**
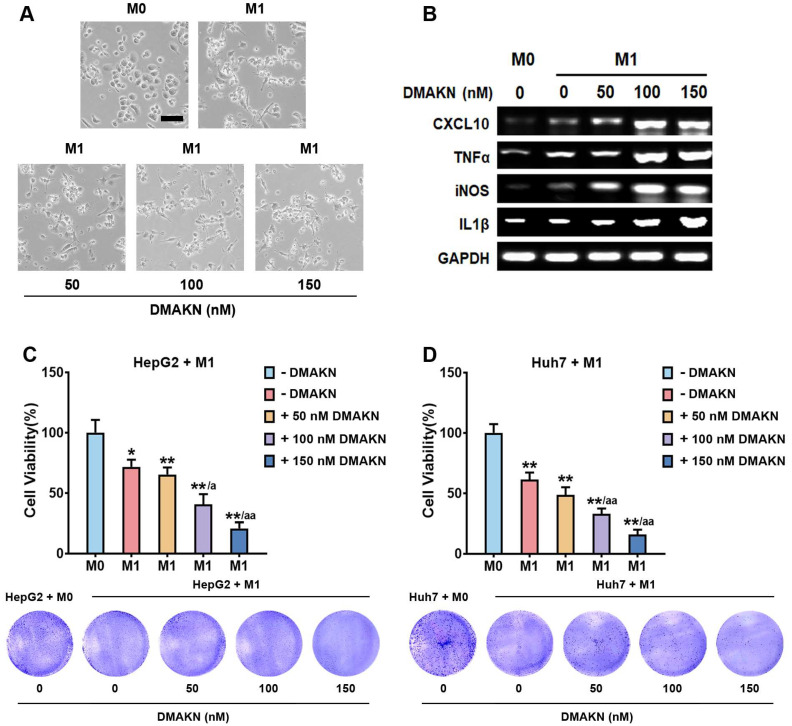
DMAKN enhances M1 macrophage polarization. (**A**) Morphological characteristics of M1 macrophages with addition of DMAKN. Scale bar = 50 μm. (**B**) mRNA expression of M1 markers in DMAKN-enhanced M1 macrophages. (**C**) Co-culture of DMAKN-enhanced M1 macrophages with HepG2 and (**D**) Huh7 cells. upper panel: measurement of HCC cell viability. lower panel: visualization of formed formazan crystals in co-cultured plates. * *p* < 0.05, ** *p* < 0.01, compared with M0 cells. ^a^ *p* < 0.05, ^aa^ *p* < 0.01, compared with M1 cells without DMAKN.

After confirming DMAKN’s ability to induce M1 macrophage polarization, we investigated its effect on polarization induced by LPS/IFN-γ. DMAKN was added to M0 cells along with LPS/IFN-γ. Following 3 d of treatment, DMAKN intensified the typical M1 macrophage morphology induced by LPS/IFN-γ (Figure 4A). To validate these observations, we assessed M1 markers expressions. Figure 4B shows that while LPS/IFN-γ increased markers expressions, co-treatment with DMAKN further enhanced makers expressions in a dose-dependent manner, accelerating M1 polarization.

Next, we evaluated the impact of DMAKN-enhanced M1 macrophages on HCC cells. Co-culturing DMAKN-enhanced M1 macrophages with HCC cells revealed the significant inhibition of cell viability (Figure 4C,D). Notably, DMAKN concentrations of 100 and 150 nM resulted in greater inhibition compared to LPS/IFN-γ, underscoring DMAKN’s potent anti-HCC activity. These results confirm that DMAKN enhances M1 macrophage polarization induced by LPS/IFN-γ, thereby enhancing its anti-HCC effects.

### 2.5. DMAKN Inhibits HCC Cell Viability by Suppressing M2 Macrophage Polarization

**Figure 5:**
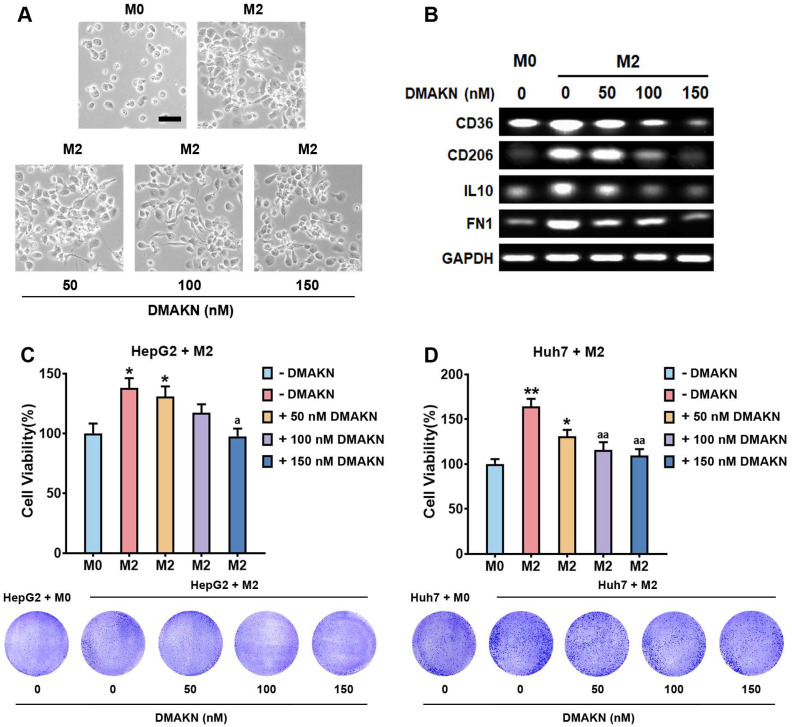
DMAKN suppresses M2 macrophage polarization. (**A**) Morphological characteristics of M2 macrophages with addition of DMAKN. Scale bar = 50 μm. (**B**) mRNA expression of M2 markers in DMAKN-suppressed M2 macrophages. (**C**) Co-culture of DMAKN-suppressed M2 macrophages with HepG2 and (**D**) Huh7 cells. Upper panel: measurement of HCC cell viability. Lower panel: visualization of formed formazan crystals in co-cultured plates. * *p* < 0.05, ** *p* < 0.01, compared with M0 cells. ^a^ *p* < 0.05, ^aa^ *p* < 0.01, compared with M2 cells without DMAKN.

We further investigated DMAKN’s impact on M2 macrophage polarization using IL-4/ IL-3 to induce differentiation from M0 macrophages. Typically, M2 macrophages display elongated morphology; however, the addition of DMAKN during this process reduced this morphology, causing the maintenance of a round or oval shape akin to M0 cells and thus suggesting that DMAKN suppresses M2 polarization (Figure 5A). To confirm this effect, we assessed M2 marker expression (CD36, CD206, IL-10, FN1). Compared to M0, IL-4/IL-3-induced M2 cells showed up-regulated marker expression, which DMAKN suppressed dose-dependently (Figure 5B).

Subsequently, we co-cultured DMAKN/IL-4/IL-13-induced M2 macrophages with HCC cells and evaluated cell viability (Figure 5C,D). The elevated HCC cell viability induced by M2 macrophages was attenuated with DMAKN addition. Notably, cells treated with 100 and 150 nM DMAKN exhibited significantly reduced viability compared to those induced by IL-4/IL-13 alone. These findings underscore DMAKN’s role in inhibiting M2 macrophage polarization and its potential to suppress cell growth in HCC.

## 3. Discussion

TAMs play a crucial role in the complex cellular interactions within the TME, impacting various stages of HCC, from initiation to progression and metastasis. Their multifaceted relationship with HCC includes contributions to tumor growth, spread, immune response modulation, and metabolic pathway alterations [17]. Understanding the role of TAMs in HCC is essential for the development of novel therapeutic strategies. Targeting TAMs or their signaling pathways may disrupt their supportive role in tumor progression, offering new avenues for HCC treatment [18].

Macrophages exhibit remarkable plasticity, leading to two distinct phenotypes—M1 and M2. M1 macrophages generally exhibit anticancer properties, while M2 macrophages often promote cancer progression. TAMs, abundant in most malignant tumors, typically show an M2 profile [19]. Thus, many anticancer strategies targeting macrophages aim to inhibit or reduce M2 polarization or increase M1 polarization to exert anticancer effects [20].

Natural products have been a rich source of bioactive compounds with therapeutic potential. Many natural products exhibit strong anticancer activity by inhibiting proliferation, inducing cell death, and preventing metastasis. Recent research has found that many natural products can also regulate TAMs and exert anticancer activity. For example, curcumin [21], resveratrol [22], and ginsenosides [23] have been reported to activate macrophages, enhancing their phagocytic activity and promoting M1-like phenotypes.

DMAKN is a naturally occurring naphthoquinone from various plant sources and has recently gained attention for its potential anticancer properties [24]. Studies have shown its ability to induce cell cycle arrest and apoptosis in colorectal cancer cells by targeting FGFR [25]. However, its effect on TAM modulation remains unclear. Thus, this study investigated DMAKN’s modulation of TAMs and its anti-HCC activity.

In our study, we utilized THP-1 cells, a human monocytic cell line, as an in vitro model to study the behavior and functions of TAMs modulated by DMAKN [16]. We successfully induced THP-1 cells into M1-polarized macrophages using LPS/IFN-γ, as well as into M2-polarized macrophages using IL-4/IL-13. For M1 macrophages, we selected CXCL10, TNF-α, iNOS, and IL-1β as markers, while FN1, CD36, CD206, and IL-10 were used as markers for M2 macrophages. Co-culturing these macrophages with HCC cell lines (HepG2 and Huh7) confirmed that M1-polarized macrophages inhibit HCC cell growth, whereas M2-polarized macrophages promote it.

To investigate DMAKN’s ability to regulate macrophages, we first determined its non-toxic concentration. Our screening showed that DMKAN concentrations below 150 nM did not affect the viability of THP-1, M0, M1, or M2 cells, even after 72 h, indicating its non-toxicity at these levels. Therefore, we used this concentration in subsequent experiments. We examined DMAKN’s effect on macrophage polarization by adding it to already differentiated M0 cells and observed morphological changes similar to those of the M1 cells. Analysis of the M1 phenotype marker revealed an increased expression after DMAKN addition, confirming its ability to induce M1 polarization. Current studies indicate that the regulation of macrophage M1 polarization is highly complex, involving multiple signaling pathways [26]. While our results demonstrated that DMAKN has the ability to regulate M1 macrophage polarization, the specific mechanism of action requires further investigation. Co-culturing DMAKN-induced M1 macrophages with HCC cells significantly reduced HCC cell viability, further verifying DMAKN’s anti-HCC effect via M1 polarization.

Additionally, we investigated the effect of DMAKN during LPS/IFN-γ-induced M1 polarization. Adding DMAKN increased M1 morphological characteristics and enhanced M1 marker expression. Co-culturing these macrophages with HCC cells showed that DMAKN enhanced the inhibitory effect of LPS/IFN-γ-induced M1 macrophages on HCC cell viability, demonstrating DMAKN’s ability to enhance M1 polarization.

**Figure 6:**
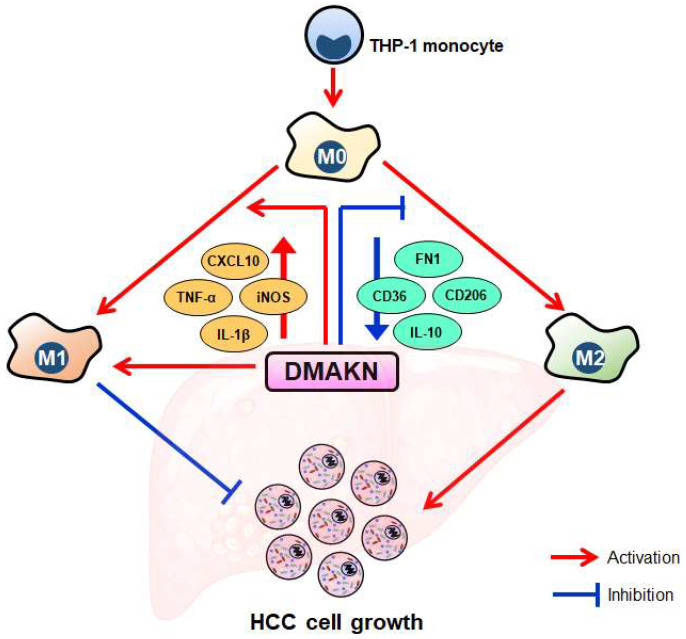
Conceptual overview of DMAKN’s influence on macrophage polarization and its impact on HCC cell growth. DMAKN induces M1 polarization and enhances M1 polarization induced by LPS/IFN-γ. Additionally, DMAKN suppresses M2 polarization induced by IL-4/IL-13. The modulation of DMAKN on both M1 and M2 macrophages inhibits HCC cell growth.

Conversely, adding DMAKN to the IL-4/IL-13-induced M2 macrophage polarization system decreased the elongated morphology of M2 macrophages and reduced M2 marker expression. Co-culturing these M2 macrophages with HCC cells showed that DMAKN inhibited their ability to promote HCC cell viability, demonstrating that DMAKN inhibits M2 polarization. The overall results of this study are summarized in Figure 6. Our results fully demonstrate that DMAKN can induce macrophage M1 polarization, enhance the ability of LPS/IFN-γ to induce M1 polarization, and weaken the ability of IL-4/IL-13 to induce M2 polarization. However, the specific signaling pathways through which DMAKN mediates these effects remain unclear. Is DMAKN acting through a signaling pathway, or are multiple pathways involved in its ability to induce M1 polarization, enhance LPS/IFN-γ-induced M1 polarization, or weaken IL-4/IL-13-induced M2 polarization? These questions are important scientific issues that warrant further exploration in the next stage of our research.

## 4. Materials and Methods

### 4.1. Reagents

β,β-dimethylacrylalkannin (DMAKN), phorbol 12-myristate 13-acetate (PMA), and thiazolyl blue (MTT) were obtained from MedChemExpress (Shanghai, China). Lipopolysaccharide (LPS), interferon-gamma (IFN-γ), interleukin-4 (IL-4), and interleukin-13 (IL-13) were obtained from Servicebio (Wuhan, China). Using the compound preparation methods in cell-based experiments [27], stock solutions of DMKAN (10 mM), PMA (1.5 mM), LPS (100 μg/mL) were prepared in DMSO. Stock solutions of IFN-γ (20 μg/mL), IL-4 (20 μg/mL), and IL-13 (20 μg/mL) were prepared in DEPC water. For the experiments, the reagents were added to the system at the required working concentrations by taking the appropriate volumes from the stock solutions. The concentration of DMSO in each step was maintained at less than 0.1%, a level shown through extensive experiments to have no effect on the secretion and polarization of THP-1 cells [28,29,30]. 

### 4.2. Cell Culture

The THP-1, HepG2, and Huh7 cell lines were obtained from ATCC. THP-1 cells were cultured in an RPMI 1640 medium supplemented with 10% FBS at 37 °C with 5% CO_2_. HepG2 and Huh7 cell lines were cultured in DMEM supplemented with 10% FBS at 37 °C with 5% CO_2_.

### 4.3. The Differentiation, Polarization, and Co-Culture of THP-1 Macrophages with HCC Cells

Following the method described in the report [31], the THP-1 cells were seeded at 8 × 10^4^ cells/well in a 24-well insert and cultured for 24 h in RPMI 1640 medium. Subsequently, the cells were differentiated into a macrophage-like phenotype (M0) by adding PMA (150 nM) and incubating for another 24 h. M0 macrophages were then polarized into M1 macrophages by adding LPS (100 ng/mL) and IFN-γ (20 ng/mL) for 72 h, or into M2 macrophages by adding IL-4 (20 ng/mL) and IL-13 (20 ng/mL) for the same duration. To study the DMAKN-modulated macrophage polarization, DMAKN was applied to M0 alone or in combination with M1 or M2 inducers for 72 h. After this period, the inserts containing differentiated macrophages were transferred onto HCC cells, which had been pre-plated into 24-well plates, for co-culture to analyze the effect on the HCC cells.

### 4.4. MTT Assay for Screening DMAKN Concentrations

The screening of DMAKN concentrations for macrophages was conducted using the MTT assay. The THP-1 monocytic cells or differentiated macrophages were initially seeded at a density of 5 × 10^3^ cells/well in 96-well plates 24 h before DMAKN treatment. The evaluation of DMAKN’s influence on HCC cell viability was performed at various concentrations and time points.

### 4.5. Analysis of the Impact of Macrophages on HCC Cells

The impact of macrophages on HCC cells was analyzed through co-culture. HCC cells were pre-seeded at a density of 1.6 × 104 cells/well in 24-well plates. Macrophages were then co-cultured with HCC cells at a 5:1 ratio. After 48 h of co-culture, the inserts were moved, and MTT solution was added to each well, incubating for 4 h to form formazan crystals. The supernatant was removed, and the crystals in each well were photographed and recorded. The crystals were then dissolved with 200 µL of DMSO, and absorbance was measured at 570 nm using a microplate reader.

### 4.6. Reverse Transcriptase (RT)—Polymerase Chain Reaction (PCR) Analysis

Total RNA was extracted from the cells using Trizol reagent and quantified with a NanoDrop Lite spectrophotometer (Thermo). From the isolated total RNA (2 µg) of THP-1-differentiated macrophages and TAMs, first-strand cDNA was synthesized using oligo dT. This cDNA served as the template for PCR amplification with G-Taq polymerase. Specific primers for M1 and M2 polarization markers were used for the PCR assay, with primer sequences provided in Table 1. The PCR protocol involved initial denaturation at 94 °C for 5 min, followed by 35 cycles of 94 °C for 30 s, 55–60 °C for 30 s, and 72 °C for 30 s. The process concluded with a final extension step at 72 °C for 10 min. The amplified PCR products were then separated on a 1.5% agarose gel stained with ethidium bromide, allowing for visualization.

### 4.7. Statistical Analysis

The data were presented as mean ± standard errors with three independent experiments. Statistical significance was determined using one-way analysis of variance (ANOVA) using GraphPad Prism 8.0 software. Data are taken as significance when *p* < 0.05.

## 5. Conclusions

In summary, our study established a THP-1 macrophage model and a co-culture model with HCC cells to examine DMAKN’s regulation of macrophages. Our results demonstrate that DMAKN not only induces M1 polarization but also enhances M1 polarization induced by LPS/IFN-γ. Furthermore, DMAKN suppresses M2 polarization of macrophages. Its modulation on TAMs inhibits HCC cell growth. These findings suggest that DMAKN can effectively regulate the TME in HCC, although further experiments are needed for verification.

## Figures and Tables

**Table 1:** Primer sequences used for PCR.

Gene Name	GenBank Acc. No.	Primer Sequences (5′-3′)	Expected Size (bp)
CXCL10	NM_001565.4	Sense: CCACGTGTTGAGATCATTGCT	152
		Antisense: TGCATCGATTTTGCTCCCCT	
TNF-α	NM_000594.4	Sense: AGCCCATGTTGTAGCAAACC	260
		Antisense: GGCTCTTGATGGCAGAGAGG	
iNOS	NM_000625.4	Sense: GGCAAGCCCAAGGTCTATGT	187
		Antisense: CCTCGACCTGCTCCTCATTC	
IL-1β	NM_000576.3	Sense: AACCTCTTCGAGGCACAAGG	197
		Antisense: GTCCTGGAAGGAGCACTTCAT	
FN1	NM_001306129.2	Sense: CCGCCGAATGTAGGACAAGA	261
		Antisense: GACAGAGTTGCCCACGGTAA	
CD36	NM_000072.3	Sense: GGTCCTTATACGTACAGAGTTCG	164
		Antisense: GCCACAGCCAGATTGAGAAC	
CD206	NM_002438.4	Sense: GTGATGGGACCCCTGTAACG	111
		Antisense: CTGCCCAGTACCCATCCTTG	
IL-10	NM_000572.3	Sense: AAGAAGGCATGCACAGCTCA	249
		Antisense: GGCAACCCAGGTAACCCTTA	
GAPDH	NM_014364.5	Sense: TGTGGGCATCAATGGATTTGG	116
		Antisense: ACACCATGTATTCCGGGTCAAT	

## Data Availability

The data presented in this study are available from the corresponding author upon reasonable request.
